# Stem- and Progenitor Cell Proliferation in the Dentate Gyrus of the Reeler Mouse

**DOI:** 10.1371/journal.pone.0119643

**Published:** 2015-03-11

**Authors:** Mirjam Sibbe, Emanuel Kuner, Daniel Althof, Michael Frotscher

**Affiliations:** 1 Center for Neuroscience, Institute of Anatomy and Cell Biology, University of Freiburg, Albertstr. 23, D-79104, Freiburg, Germany; 2 Institute of Physiology, University of Freiburg, Hermann-Herder-Str. 7, D-79104, Freiburg, Germany; 3 Institute for Structural Neurobiology, Center for Molecular Neurobiology Hamburg, Falkenried 94, D-20251, Hamburg, Germany; University of Victoria, CANADA

## Abstract

Adult hippocampal neurogenesis has been implicated in hippocampus-dependent learning and memory. Furthermore, the decline of neurogenesis accompanying aging could be involved in age-related cognitive deficits. It is believed that the neural stem cell niche comprises a specialized microenvironment regulating stem cell activation and maintenance. However, little is known about the significance of the extracellular matrix in controlling adult stem cells. Reelin is a large glycoprotein of the extracelluar matrix known to be of crucial importance for neuronal migration. Here, we examined the local interrelation between Reelin expressing interneurons and putative hippocampal stem cells and investigated the effects of Reelin deficiency on stem cell and progenitor cell proliferation. Reelin-positive cells are found in close vicinity to putative stem cell processes, which would allow for stem cell regulation by Reelin. We investigated the proliferation of stem cells in the Reelin-deficient reeler hippocampus by Ki67 labeling and found a strong reduction of mitotic cells. A detailed analysis of dividing Type 1, type 2 and type 3 cells indicated that once a stem cell is recruited for proliferation, the progression to the next progenitor stage as well as the number of mitotic cycles is not altered in reeler. Our data point to a role for Reelin in either regulating stem cell quiescence or maintenance.

## Introduction

Adult hippocampal neurogenesis plays an important role for hippocampus-dependent learning and memory [1, 2]. Further, evidence is accumulating that underlines a crucial role of adult neurogenesis in aging-related cognitive deficits and in depression [3, 4, 5, 6]. Conceptually, the stem cell niche controls maintenance and activation of the residing stem cells. The proximity of the niche to vasculature, glial cells and neurons is believed to keep stem cells under control by emitted vascular factors, paracrine or membrane-tethered factors and transmitters [7, 8, 9, 10, 11]. The extracellular matrix composed of large, often highly glycosylated molecules could play a role in regulating stem cells by either anchoring signaling factors thus leading to their accumulation or modulated stability and additionally by activating signaling pathways. Reelin is an extracellular matrix molecule whose importance for migration and synaptic integrity is well discerned. The lack of Reelin in the reeler mutant leads to severe defects, including a principally inversed cortical layering, dispersion of hippocampal granule cells, cerebellar hypoplasia and synaptic impairment [12, 13, 14, 15, 16, 17, 18]. Importantly, Reelin signaling has been found to be essential for the development of a normal hippocampal radial glial scaffold [19]. Radial glia are neurogenic during development and are thought to constitute the radial stem cells of the adult hippocampus [20, 21, 22]. Moreover, Reelin has previously been implicated in the regulation of adult hippocampal neurogenesis [23, 24]. The reeler mutant exhibited a decreased number of newly generated, Doublecortin-positive (DCX+) hippocampal neurons [25]. The underlying mechanisms remain to be elucidated. We aimed to find out whether Reelin regulates proliferation or differentiation of neuronal stem cells and progenitor cells. We therefore analyzed Ki67-expressing (Ki67+), dividing cells along the putative neuroprogenitor lineage and examined the morphological relation between Reelin-positive (Reelin+) cells and stem cells. Our analysis did not provide evidence for effects of Reelin on stem cells that have already entered the cell cycle. The data rather suggest a role for Reelin in controling stem cell quiescence or, alternatively, in the proper organization of the stem cell niche during development.

## Material and Methods

### Animals

*Reeler* mutant mice (B6C3Fe strain) were identified by their well-known morphological malformations in the cortex and hippocampus. The genotypes of the mutants were confirmed by PCR analysis of genomic DNA, as described previously [26]. Only males, aged 3 weeks were included into the study. Mice were housed in groups under standard conditions (23 ± 1°C, 40–50% humidity, food and water *ad libitum*) with an inverted 12:12 light:dark cycle (light off at 07:00 AM). All experiments were performed in accordance with the guidelines of the European Communities Council Directive of 22 September 2010 (2010/63/EU) and approved by the regional council approved by the Institutional Animal Care and Use Committee Center for Experimental Models and Transgenic Services-Freiburg (CEMT-FR) as well as the regional council of Freiburg.

### Immunohistochemistry

Tissue preparation. Three-weeks old mice were deeply anesthetized with Narkodorm-n (Pentobarbital; 180 mg/kg, i.p.; Alvetra, Neumünster, Germany). For light microscopic immunohistochemistry animals were perfused transcardially with a fixative solution containing 4% paraformaldehyde (Merck, Darmstadt, Germany) in 0.1 M phosphate buffer (PB, pH 7.2) and postfixed for 2h. For electron microscopic immunohistochemistry, the fixative additionally contained 15% (v/v) saturated picric acid and 0.05% glutaraldehyde (Polyscience). Brains were sectioned on a vibratome at 50 μm in the coronal plane.

### Light microscopy

Sections were blocked in 10% donkey serum in PB and incubated with antibodies detecting glial fibrillary acidic protein (GFAP, rabbit polyclonal, 1:1000, DAKO, Glostrup, Denmark or mouse monoclonal, mGFAP, 1:1000, Sigma-Aldrich, Taufkirchen, Germany), Ki67 (rabbit monoclonal, 1:1000, Novocastra, Wetzlar, Germany), Nestin (chicken polyclonal, 1:500, Abcam, Cambridge, UK) and Doublecortin (DCX, goat polyclonal, 1:1000, Santa Cruz Biotechnology Inc., Santa Cruz, CA, USA). For visualization of immunostaining, Alexa-conjugated fluorescent secondary antibodies (1:500, Dianova, Hamburg, Germany) were used. Sections were coverslipped with fluorescent mounting medium (DAKO, Glostrup, Denmark).

### Immunoelectron microscopy

For double immunoelectron microscopy, sections were cryoprotected and freeze-thawed. After blocking in 20% NGS in 50 mM TBS, sections were incubated with primary antibodies for GFAP (rabbit, polyclonal, DAKO) and Reelin (mouse-monoclonal, clone G10, Millipore, Billerica, MA, USA) in 50 mM TBS containing 3% NGS (Vector Laboratories, Burlingame, CA) for 24h at 4°C. After washes in TBS, the sections were incubated with biotinylated goat anti-mouse IgG antibody (1:100; Vector Laboratories) and goat anti-rabbit (Fab fragment, 1:100) coupled to 1.4 nm gold (Nanoprobes, Stony Brook, NY). Subsequently, sections were processed for silver enhancement of the gold particles with an HQ Silver kit (Nanoprobes) and incubated with avidin-biotin peroxidase complex (ABC kit; Vector Laboratories) that was visualized with 3,3′-diaminobenzidine tetrahydrochloride (0.05%) as a chromogen and 0.01% H_2_O_2_ as substrate. Sections were then treated with 1% osmium tetroxide and uranyl acetate, dehydrated and flat-embedded in epoxy resin (Durcupan ACM Fluka; Sigma-Aldrich). Ultrathin sections were cut at 60–70 nm on an ultramicrotome (Reichert Ultracut E; Leica), and viewed on a Philips CM100 electron microscope. Images were taken with a CCD camera (Orius SC600; GATAN) and analyzed using GATAN imaging software.

### Quantitative analysis of immunostainings

For quantitative analysis of fluorescently labelled sections, images were captured using a confocal laser-scanning microscope (LSM 510, Carl Zeiss, Jena, Germany), equipped with an Argon laser (excitation lines: 458, 488 and 514) and a He-Ne laser (excitation lines: 543 and 635 nm). To image the area of the dentate gyrus, a 10x objective was used; for visualisation of immunostained cells a Plan-Neofluar 40xI 1.3 oil objective was used. The coordinates corresponded to Bregma −1.2 to −2.2. Due to the granule cell dispersion found in the reeler hippocampus, a subgranular zone (SGZ) cannot be defined. Therefore, the entire dentate gyrus was included into the analysis in control and reeler animals. For the evaluation of densities of Ki67+ cells, all Ki67+ cells within the dentate gyrus per image were counted; the area of the dentate gyrus was measured by tracing the dentate gyrus using ImageJ, and the ratio of Ki67+ cells per area calculated. The mean value per animal was acquired from all section images. For evaluating the proportions of type1, type 2, and type 3 cells among all Ki67+ cells, the percentage of each type per counted total of Ki67+ cell was calculated per animal.

Quantitative analysis was performed using ImageJ (http://rsbweb.nih.gov/ij/index.html). Sections containing the dorsal hippocampus from 3 reeler mice, 3 heterozygous and 3 wild-type animals were used for acquisition of Ki67+ cell numbers. Sections for the analysis of type 1, 2, 3 cells derived from 3 reeler mutants and 2 control animals. Using the marker combination Ki67/Nestin/GFAP, a total of 144 control and 310 reeler Ki67+ cells were analyzed. For the combination Ki67/Nestin/DCX a total of 274 control and 202 reeler cells were used.

### Statistical analysis

Statistical analysis was done using GraphPad InStat 3.0 (GraphPad Software, Inc., San Diego, CA). Student’s t-test and ANOVA were employed. Statistical significance was assumed with *p* ≤ 0.05. Values are expressed as mean ± SEM.

## Results

**Fig 1:**
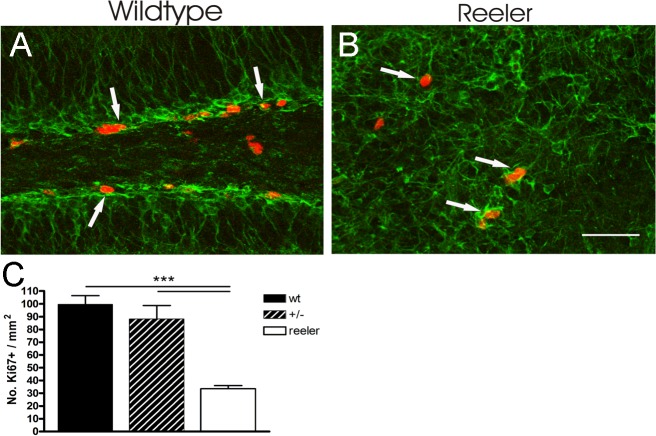
Reduced proliferation in reeler hippocampus. **A, B** show an example of Ki67+ mitotic cells (red, arrows) of the hippocampal dentate gyrus in a wild-type mouse (A) and a reeler mouse (B). Additional labeling of young DCX+ (green) neurons reveals the structural disorganization in *reeler*. **C** Quantification of Ki67+ cells. There are significantly reduced numbers of mitotic cells in the *reeler* dentate gyrus. Mean values + SEM are given, *** p < 0.001. Scale bar: 50 μm. wt: wild-type, GCL: granule cell layer, SGZ: subgranular zone, H: hilus.

To find out whether Reelin plays a role in regulating stem cell proliferation we analyzed the number of dividing cells positive for Ki67, a protein present during all active phases of the cell cycle, in the dentate gyrus of Reelin—deficient reeler mice in comparison to control animals (Fig. 1). Whereas in control animals 99.6 ± 6.8 cells/mm^2^ underwent cell cycling, in reeler animals only 33.5 ± 2.5/mm^2^ Ki67—positive (Ki67+) cells were found. The results were statistically significant (ANOVA, p ≤ 0.0001, F = 22.8, post-hoc Tukey's Multiple Comparison Test, p ≤ 0.001). With 88.2 ± 10.5/mm^2^ dividing cells in heterozygous animals, numbers were comparable to wild-type mice.

**Fig 2:**
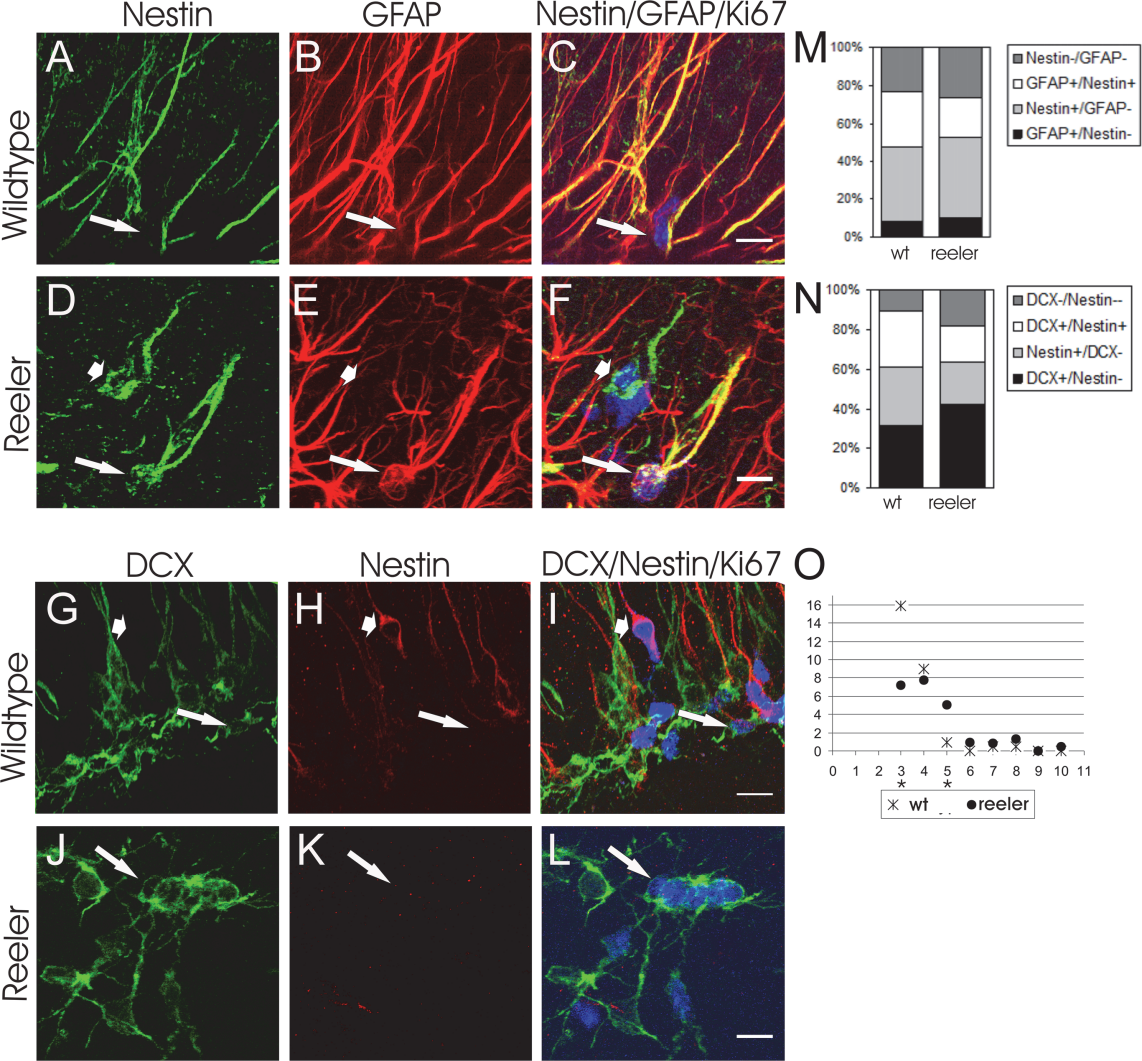
Type 1, type 2 and type 3 cells undergoing mitosis. **A-C** show an example of a Nestin+/GFAP+/Ki67+ type 1 cell in the control dentate gyrus (arrow: Ki67, blue), **D-F** in the *reeler* dentate gyrus (arrow: Ki67). In D-F additionally a Nestin+/GFAP−/Ki67+ is labelled (short arrow). **G-I** display a DCX+/Nestin−/Ki67+ type 3 cell (arrow) in a wild-type animal, **J-L** in *reeler*. In **G-I** an additional DCX-/Nestin+/Ki67+, presumptive type 1 cell is present (short arrow). **M, N** Quantified cell fractions among all mitotic cells in the dentate gyrus for the two triple-staining experiments performed. **O** Incidence of Ki67+ cluster size in control and *reeler* dentate gyrus. In control animals cell clusters often consisted of only three cells, whereas clusters in *reeler* contained significantly more, often five cells. Data are represented by mean values; * p < 0.05, *** p < 0.0001. Scale bars: 10 μm.

Within the hippocampal stem cell niche a heterogeneous population of stem- and progenitor cells reside. According to a classification proposed by Kempermann et al. [27] the Nestin− as well as GFAP− positive, putative type 1 stem cells can be discriminated from Nestin+/GFAP− type 2a and Nestin+/DCX+ type 2b cells. Type 3 cells finally exhibit DCX but have lost Nestin expression. Does the reduced proliferation observed in reeler mutants result from Reelin influencing proliferation or differentiation of a certain type of stem cell or progenitor cell? To analyze this, the numbers of stem cells and progenitor cells among Ki67+ dividing cells were determined using Ki67 labeling in combination with GFAP and Nestin or Ki67 in combination with Nestin and DCX (Fig. 2). In reeler animals 20.4 ± 4.0% of Ki67+ cells were Nestin+/GFAP+, therefore could be classified as type 1 cells. 42.9 ± 3.6% were Nestin+/GFAP− type 2, and 42.6 ± 7.4% DCX+/Nestin− type 3 cells. It should be noted that these values derive from two different labeling experiments. The number of type 1, type 2 and type 3 cells in control animals were 28.9 ± 1.9%, 39.2 ± 2.7 and 30.9 ± 0.5%, respectively, and did not differ significantly from reeler animals (Student’s t-test, p > 0.05).

Conspicuously, in the reeler mutant Ki67+ proliferating cells more often were found in larger cell clusters (Fig. 2). By analyzing the occurrence of Ki67+ cells in relation to cell cluster size we indeed found enhanced attachment, respectively a shift to higher cell numbers per cluster in Reelin deficient animals. Whereas in control animals 15.90 ± 4.0% Ki67+ cells were found in small clusters consisting of only 3 cells, 7.18 ± 1.3% Ki67+ cells were located in clusters of 3 in reeler animals (Student’s t-test, p ≤ 0.05). To the contrary, only 0.90 ± 0.5% of Ki67+ cells were found in larger clusters of 5 in control animals compared to 5.05 ± 0.2% in Reelin deficient mice (Student’s t-test, p ≤ 0.0001).

**Fig 3:**
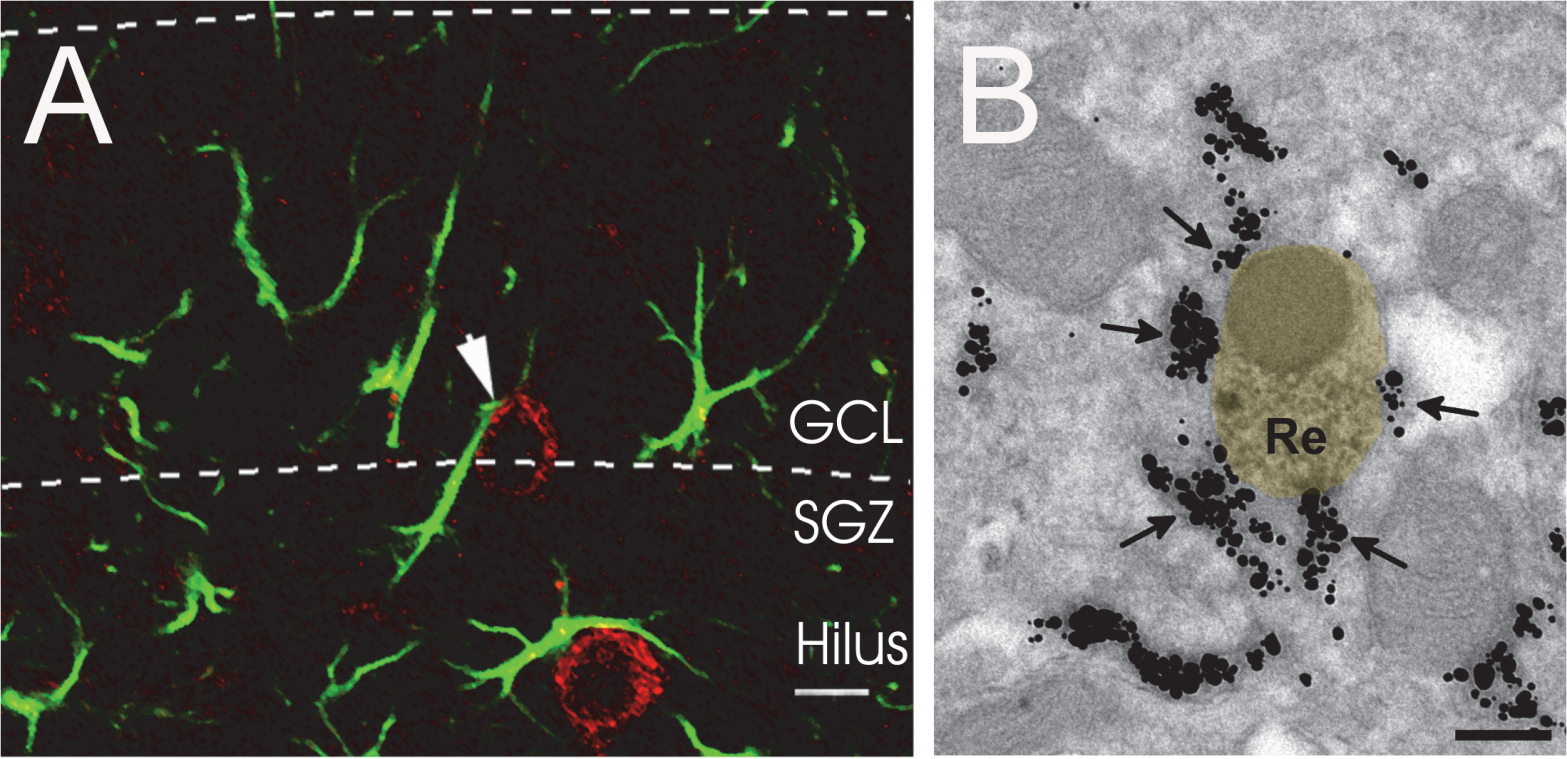
A Close proximity between a Reelin+ interneuron (red) and a GFAP+ putative stem cell (green) in the SGZ of a wild-type mice. **B** Example of close apposition of GFAP+ glial processes and a Reelin+ dendrite as revealed by electron microscopic double immunolabeling. Reelin labeling is visualized by DAB precipitate (yellow overlay), GFAP by gold particles (arrows). Scale bar in A: 10 μm, in B: 0.2 μm. GCL: granule cell layer, SGZ: subgranular zone.

The major source of Reelin in the adult brain are Parvalbumin-expressing interneurons [28,29]. We further aimed to elucidate the morphological relationship of Reelin-expressing interneurons and GFAP+ stem cells of the SGZ. Using immunohistochemistry we could detect Reelin+ interneurons in close apposition to GFAP+ radial-oriented putative stem cells of the SGZ (Fig. 3A). Furthermore, in electron microscopic analysis GFAP+ and Reelin+ processes could be found in close apposition in the SGZ which is supporting a possible regulation of postnatal stem cells by Reelin secreted from interneurons (Fig. 3B). Measuring the extent of Reelin+ membrane covered by GFAP+ profiles resulted in 32.4 ± 3.2% coverage (from a total of nine examples).

## Discussion

In this study we aimed at gaining insight into a regulatory role of Reelin in stem cell or neuronal progenitor proliferation and differentiation. Analysis of proliferating Ki67+ cells in *reeler* mutants, heterozygous animals, and wild-type mice revealed a dramatic reduction of proliferation in the dentate gyrus of Reelin-deficient *reeler* mice in comparison to both, heterozygous and wild-type animals. We are confident that these findings reflect an effect of Reelin on the stem cell niche although no detailed stereological analysis was performed. Our results confirm and extend previous studies [25] and suggest that altered proliferation is the cause of the reduced numbers of newly generated DCX+ cells 15 days after the first BrdU injection, as reported [25]. In heterozygous animals intermediate levels of Reelin expression were found [30]. Apparently in contrast to a previous study [31], in our experimental paradigm this lower amount of Reelin in heterozygous animals was sufficient to maintain a normal stem cell niche. Similarly, neuronal layering of the cortex, hippocampus and dentate gyrus and the vertical arrangement of the dentate radial glial scaffold, which are distorted in *reeler*, develop normally in heterozygous animals [19,32].

The adult hippocampal stem cell niche consists of a heterogeneous group of putative stem- and progenitor cells that at least in part can be identified by application of different marker combinations. If and how these stem cell/progenitor types are specifically regulated is not known. In order to find out about a potential role of Reelin in influencing proliferation or differentiation of type 1, type 2 or type 3 stem cells, we determined the numbers of Ki67+/Nestin+/GFAP+ type 1 cells, Ki67+/Nestin+/GFAP− type 2 cells and Ki67+/Nestin−/DCX+ type 3 cells. Since the lack of Reelin has been described to influence cell polarity, the orientation of cells could not reliably be used as acriterion. We regard it as a main finding of the present study that the ratios of type 1, type 2, and type 3 cells among all proliferating cells were not significantly different between genotypes. However, in view of the low number of animals available to us, and relatively high standard deviations we cannot exclude that a type II error may have occurred and that we missed significant differences. We can conclude at least that Reelin deficiency did not alter cell cycling or the differentiation of specific stem cell/progenitor cell types in a range that would reflect the 66% difference in the density of Ki67+ cells between wild-type mice and *reeler* mutants. Since cell death has been reported previously to be not enhanced in the reeler SGZ [25], the observed reduction of proliferating cells to one-third in *reeler* may likely be due to either i) reduced numbers of quiescent stem cells being recruited for proliferation or ii) a developmentally disrupted stem cell niche not sufficiently differentiated to maintain normal stem cell numbers. Which of these alternatives holds true, needs to be addressed in future experiments by analyzing inducible knock-out mice. One finding in support of a potential function of Reelin in controlling the stem cell niche is the close proximity of Reelin+ and GFAP+ cells and processes in the SGZ.

Finally, we found a higher number of proliferating cells in *reeler* that formed clusters of 5 or more cells. Reelin has been previously discussed as a detachment signal for migrating neurons [33], and our result would support such a hypothesis. Furthermore, feedback signaling from adult generated neurons to progenitor stages has been reported [34, 35]. The reduced ability of newly generated cells to leave their niche could also influence stem cell behavior and may have contributed to the reduction in proliferation we observed in r*eeler*.

In summary then, our data indicate a role for Reelin either in developing, maintaining or regulating type 1 stem cell activation. Reelin deficiency, in turn, results in reduced adult-generated neurons in the *reeler* mutant [25]. Furthermore, Reelin signaling has been shown to be crucial for normal dendritic maturation of adult-generated neurons [36]. The importance of adult hippocampal neurogenesis for learning and memory processes has been demonstrated [1,2], and impaired adult neurogenesis might play a role in deficits accompanying aging as well as depression [3, 4, 5, 6]. Along this line, Reelin signaling was found altered in several neurological disorders including depression, bipolar disorder, schizophrenia and autism [37, 38, 39,40]. We hypothesize Reelin to be an essential player in normal brain function by controlling adult neurogenesis via recruiting quiescent stem cells for proliferation, thereby contributing to an adaptation to environmental needs.
